# Supplementary material for: Phytochemicals as Chemo-Preventive and Therapeutic Agents Against Bladder Cancer: A Comprehensive Review
Source: Diseases. 2025 Mar 30;13(4):103. doi: 10.3390/diseases13040103 (PMC12026019; doi:10.3390/diseases13040103)
Supplement: Supplementary file 1 [file diseases-13-00103-s001.zip › diseases-3468635-supplementary.pdf]

	Supplementary Table S1 : Selected clinical studies regarding the effects of phytochemicals in various types of cancer					
**Study name**	**Study duration**	**Intervention (n)**	**Controls (n)**	**Type of cancer**	**Form and dose of phytochemical**	**Results**
Ryan et al. 2013 [173]	Througho ut the course of radiothera py	14	26	Breast cancer	6 g curcumin (3 times x 2 g)	↓ severity of radiation dermatitis
Belcaro et al., 2014 [174]	60 days	Chemo- radio-therapy: 80	78	Chemotherapy: colon/rectum, liver or kidney, stomach, lung, gynecological or hematological malignancies<br><br>Radiotherapy: colon/rectum, liver or kidney, stomach, or lungs	500 mg Meriva (100mg curcuminoids (ratio curcumin: demethoxycurcumin: bis-demethoxycurcumin 33:8:1)<br>1x3 times per day after meal	↓ gastrointestinal symptoms<br>↓ fatigue,<br>↓ malnutrition<br>less cognitive problems

Hejazi et al., 2016 [175]	9 weeks	20	20	Prostate cancer	3 grams of curcumin (2 capsules with each meal)<br><br>Capsule of 440 mg curcumin (curcumin of 347 mg, desmethoxycurcumin of 84 mg, and bisdesmethoxycurcumin of 9 mg) and essential oil of turmeric of 38 mg.<br><br>Note: Patients and controls received also radiation therapy.	↑ total antioxidant capacity<br>↓ superoxide dismutase<br><br>↓ PSA levels in both groups<br><br>no significant differences in treatment outcomes
Howells et al., 2019 [176]	once every 2 weeks	18	9	Metastatic colorectal cancer	folinic acid, fluorouracil, and oxaliplatin ± bevacizumab plus<br>2 g oral Curcumin C3 Complex/d (~80% curcumin and 20% demethoxycurcumin and bisdemethoxycurcumin)	↑ overall survival in intervention group<br><br>No change in progression-free survival, quality of life or neurotoxicity<br><br>No difference in chemokine (C-X-C motif) ligand 1
Hidayat et al., 2021 [177]	7 days	20	20	Cervical cancer	4 g curcumin/day<br><br>Note: Patients and controls received also radiation therapy.	↑ radiosensitivity (↑ survivin levels) in the intervention group
Chaiworramukkul et al., 2022 [178]	8 weeks	17	16	Various types with cancer anorexia–cachexia syndrome)	800 mg curcumin x 2<br>Each capsule was contained 240 mg of curcuminoids.	No changes in body composition

						Slower reductions of hand-grip strength, slower reductions in basal metabolic rate.
Gunther et al. 2022 [179]	Througho ut chemorad iation therapy and 6 weeks	15	7	rectal cancer	4 g total (x2 per day)	Curcumin did not increase pathologic complete response
Ramezani et al., 2023 [180]	21 days	13 curcumin mouthwash<br>12 curcumin capsules	12 placebo mouthwash	Head and neck cancer	Mouthwash: 100 mg of curcumin powder, freshly prepared solution (10 ml of freshly prepared mouthwashes x 3 times a day)<br>Capsule: Sinacurcumin soft gel (nano-micelles with 40 mg curcuminoids) (SinaCurcumin®40)	↓ pain and burning related to oral mucositis.<br>no difference between curcumin mouthwash and capsules
Talakesh et al., 2022 [181]	5 days per week for 5 weeks	21	21	Breast cancer under radiotherapy	Capsules of nano-curcumin (80 mg per day)	↓ severity of radiation-induced skin reactions at week 7 (no difference in previous time-points)<br><br>↓ self-reported pain
Cruz-Correa et al. 2018 [182]	12 months	21	23	Colorectal cancer	Oral curcumin 3g/day (1,5 g x 2 times per day)	
Saadipoor et al 2019 [183]	3 days before and during the radiothera py	31	33	Prostate cancer	Oral nanocurcumin (120 mg/day) or placebo (40 mgx 3 times per day)	No difference in radiation toxicities nor tumor response

Zhu et al 2020 [184]	30-38 days	20	NA	head and neck	440- 2200 μmol/L three times a day	EGCG is safe and improves oral mucositis, recommended dose is 1760μmol/L
van Die et al., 2017 [185]	12 weeks	9	11	Biochemically recurrent prostate cancer	2 tablets x2 daily<br>+ 2 capsules x2 daily broccoli<br><br>*turmeric* (Curcuma longa) rhizome extract, 25:1, 95–105% total curcuminoids; curcumin 100 mg (400 mg/day);<br>*resveratrol* from Polygonum cuspidatum extract dry concentrate, 100:1, containing min 50% resveratrol, 30 mg (120 mg/day);<br><br>*green tea* (Camellia sinensis) leaf dry concentrate, 25:1, containing min 50% polyphenols; catechins 100 mg (400 mg/day;<br><br>*broccoli* (Brassica oleracea var. italica) sprout concentrate, 20:1, equivalent to fresh sprouts 2,000 mg (8 g/day).	Pilot study<br>no difference in prostate symptoms, quality of life, anxiety, and depression.

Henning et al., 2020 [186]	4 weeks	15	16	prostate cancer	1 gram of green tea extract (830 mg of green tea polyphenols) + 800 mg of Quercetin<br><br>two capsules of green tea extract (Tegreen 97) with one capsule of Quercetin (N = 15) or<br><br>two capsules of green tea extract	no effect on catechins and green tea polyphenols in prostate tissue<br><br>Quercetin may affect glucuronidation of green tea polyphenols, as demonstrated by decreased epigallocatechin and 4′-methyl epigallocatechin in the urine.
Kooshyar et al. 2017 [187]	4 weeks	10	10	on chemotherapy	250 mg quercetin hydrate (Sigma–Aldrich Co; St Louis, MO, USA)<br>x 2/ d	↓ mucositis incidence in the quercetin group, but mucositis was more severe in the intervention group, possibly due to worse oral health status
Zwicker et al. 2019 [188]	56 days	28 low dose<br>29 high dose	NA	patients with various types of cancer at high risk for thrombosis	500 mg isoquercetin<br>1000 mg isoquercetin	1000 mg isoquercetin<br>↓ 21.9% plasma D-dimer, P-selectin<br><br>no primary venous thromboembolism events or major hemorrhages

						Isoquercetin ↑protein disulfide isomerase inhibitory activity<br>↓ platelet-dependent thrombin generation
Howells et al., 2011 [189]	10 - 21 days	6	3	stage IV colorectal cancer and hepatic metastases	micronized resveratrol	↑ cleaved caspase-3 (marker of apoptosis) by 39% in malignant hepatic tissue
Zhu et al. [190]	12 weeks	12 (low dose)<br>9 (high dose)	9	women at increased risk for breast cancer	5 or 50mg trans-resveratrol twice daily for 12 weeks	↓ RASSF-1α methylation
Popat et al. 2013 [191]	4 months	24	NA	multiple myeloma	5g SRT501 +/- bortezomib	severe adverse events
Nguyen et al 2009 [192]	14 days	8	NA	colon cancer	80 and 20mg/day for	upregulated myc, axinII and Cyclin D1, downregulated CD133 and LGR5 in normal colonic tissue
Traka et al 2019 [193]	12 months	17 (intermediate)<br>16 (high)	15 (low dose)	prostate cancer	214 +/- 7.3µmol glucoraphanin vs<br>492 +/- 3.2µmol glucoraphanin vs<br>72 +/- 2.8µmol glucoraphanin<br>(glucoraphanin-rich broccoli soup)	glucoraphanin affects gene expression and reduces the risk of progression
NA: Not available						
